# An exploration of surface temperature asymmetries as potential markers of affective states in calves experiencing or observing disbudding

**DOI:** 10.1017/awf.2024.47

**Published:** 2024-11-05

**Authors:** Marco Antonio Ramirez Montes de Oca, Michael Mendl, Helen R Whay, Suzanne DE Held, Sarah L Lambton, Helena Telkänranta

**Affiliations:** 1 Animal Welfare and Behaviour Research Group, Bristol Veterinary School, University of Bristol, Langford BS40 5DU, UK; 2 NUI Galway, University Road, Galway, Ireland; 3 Arador Innovations, Kamreerintie 10, 02770 Espoo, Finland

**Keywords:** affective states, animal emotions, animal welfare, dairy cattle, infrared thermography, temperature asymmetries

## Abstract

The emotional valence hypothesis suggests an increased left-brain hemisphere activation during positive situations and *vice versa* during negative situations. Since facial thermal asymmetries may reflect lateralised brain activity, we investigated this in dairy calves subjected to hot-iron disbudding (Disbudded; n = 12) as a model of negative affective states. As affective responses can vary due to previous experiences, we examined whether calves that had (ExpObs; n = 12) and had not (InexObs; n = 12) experienced disbudding differed in their thermal response to a conspecific being disbudded, and whether calf response to the researcher (approaching, moving away, not moving) was associated with thermal asymmetries. We made thermographic recordings of each calf on three days: Day before disbudding (D1); Disbudding day (D2); and Day after disbudding (D3), and at two different times: Disbudding time/1400(T1) and Afternoon/1700(T2). Data were analysed using multilevel models. Calves had warmer left ears on D2 compared to higher temperatures on the right ear on D1, suggesting higher right-hemisphere activity on D2. ExpObs calves had higher left-eye temperatures when observing a conspecific being disbudded (D2×T1) than InexObs calves that had warmer right eyes, but this reversed on the following day (D3×T1). Calves avoiding the researcher had warmer left eyes whereas those approaching him had warmer right eyes, suggesting greater activation of the right hemisphere in the former. This study provides initial evidence of temperature asymmetries when observing or experiencing a negative event. Further work is required to confirm and build upon these early findings. The study highlights the potential for future development of methods using infrared thermography as a proxy measure of affective valence.

## Introduction

Emotional (affective) states are elicited by appraisals of an ongoing situation (Davidson *et al.* 2003), have physiological, behavioural, cognitive and subjective components (Scherer & Ekman 1984), and can be assessed in terms of valence (positive or negative) and arousal (low or high) (Russell 1980; Mendl *et al.* 2010).

Most current methods for the study of emotions in animals include interpretation of behaviour and physiological parameters. Some of these (e.g. activation of physiological stress responses) are useful for identifying arousal associated with an emotion but are less informative in terms of valence (Hinde 1985; Paul *et al.* 2005). Other methods, such as the study of cognitive bias, allow us to infer if an animal is in a more negatively or positively affective state (Mendl *et al.* 2009). However, these methods are time consuming as they require animals to be trained to perform discrimination tasks. Neural indicators of affective state are also valuable in animal studies, methods such as EEG (Davidson *et al.* 1990; Schmidt *et al.* 2001), fMRI (Canli *et al.* 1998) and near-infrared spectroscopy (Muehlemann *et al.* 2011) can be used to identify changes in brain activity, but they require equipment that is not easy to use in non-scientific or non-clinical facilities and may require the animals to remain stationary or carry equipment. For these reasons, the use of entirely non-invasive techniques that allow us to collect readings associated with physiological changes is needed to advance the study of animal emotions, including in non-laboratory settings.

Infrared thermography allows the measurement of surface temperatures in tissues or objects in real time with high accuracy, making it a potential tool for the study of thermal patterns associated with physiological changes occurring during the processing of emotions. Correlations between infrared thermography read-outs and emotions have been studied in humans (Pavlidis *et al.* 2002; Esposito *et al.* 2015) and animals (Stewart *et al.* 2008a; Kuraoka & Nakamura 2011; Proctor & Carder 2015; Lecorps *et al.* 2016). In humans, changes in temperature patterns of different regions of interest (ROIs) of the face have been associated with the arousal and valence dimension of emotions (Salazar-López *et al.* 2015). In animals, changes in peripheral temperature associated with arousal levels have been studied using infrared thermography (Stewart *et al.* 2008a,b; Kano *et al.* 2016), Stewart *et al.* found that cows showed a drop and a subsequent gradual rise in the temperature of the inner corner of the eye in response to negative handling (Stewart *et al.* 2008a), hot-iron disbudding and surgical castration (Stewart *et al.* 2008b, 2010a); changes mediated by the autonomic nervous system (Stewart *et al.* 2010b). Similarly, a drop and subsequent increase in nasal temperature associated with putative high arousal emotions have been identified in several species of animals (Kuraoka & Nakamura 2011; Proctor & Carder 2015; Kano *et al.* 2016). However, these changes are likely to occur in any high arousal emotional response regardless of emotional valence. The present study, therefore, focuses on a measure which may provide us with information regarding emotional valence by looking at asymmetries in facial temperature patterns.

Temperature asymmetries in different areas of the face may reflect asymmetries in physiological function (blood flow, muscle activation) related to lateralised brain activity (Aristizabal-Tique *et al.* 2023). For example, motor cortex activity in one brain hemisphere eliciting a muscular response in the relevant contralateral limb, eye or ear of the individual may be accompanied by temperature change due to the motor response and associated alterations in blood flow to the effector structure (Tamai *et al.* 1984; Bon & Lucchetti 1994; Montgomery *et al.* 2013). Brain lateralisation ideas posit that different types of stimuli are processed predominantly by different hemispheres of the brain. For example, mammals process spatial cues, facial recognition and novelty predominantly in the right hemisphere (Hamilton & Vermeire 1988; King & Corwin 1992; Larose *et al.* 2006), whilst processing familiarity and recognition of conspecific vocalisations predominantly in the left hemisphere (Basile *et al.* 2009). Links between brain lateralisation and affective states have also been proposed via three main hypotheses. The approach-withdrawal hypothesis states that emotions characterised by an active approach towards the stimuli (reflecting reward acquisition) are processed in the left hemisphere and emotions that result in behavioural avoidance by the individual (reflecting punishment avoidance) are processed in the right hemisphere (Davidson *et al.* 1990). A variant of the above, the emotional-valence hypothesis, suggests that negative emotions are processed more intensively in the right hemisphere and that the left hemisphere has more involvement in the processing of positive ones (Silberman & Weingartner 1986; Leliveld *et al.* 2013). Finally, the right-hemisphere hypothesis states that the majority of emotion processing takes place in the right hemisphere regardless of valence (Gainotti 1972).

It is thus possible that temperature asymmetries in different areas of the face reflect lateralised processing of emotions. Here, we provide an initial investigation of this possibility in dairy calves, focusing on several areas of the face. We hypothesised that skin temperature changes in ocular and periocular areas might reflect changes in local blood flow or muscular activity associated with motor or sensory processing in the contralateral (due to optic nerve decussation) hemisphere, e.g. preferential use of the left eye to evaluate novel stimuli indicated right-hemisphere processing (Robins & Phillips 2010). Similarly, a contralateral relationship between temperature asymmetries and brain activity is expected in the base of the ears as direct stimulation of the cortex results in the movement of the contralateral ear. On the other hand, changes in nostril and nasal passage temperatures are likely to indicate hemispheric activation on the same side of the head, as olfactory nerves project to the ipsilateral brain hemisphere (Siniscalchi *et al.* 2011). We are not aware of any studies looking at asymmetric blood flow or muscular activity in this context or their relationship with temperature asymmetries and therefore we cannot be certain of the mechanisms underlying temperature asymmetries in the areas studied.

This study measures changes in facial temperature when dairy calves are exposed to an aversive situation that likely elicits a negative emotional state – pain and restraint caused by a routine hot-iron disbudding procedure. The procedure consists of applying a hot iron (at temperatures above 500^o^C) to the ‘bud’ tissue that generates the horns of adult cows and may cause secondary damage to the surrounding tissues (Adcock & Tucker 2018). The use of local anaesthesia and analgesia for this procedure reduces the sensitivity of the area, physiological changes associated with pain (elevated heart and respiratory rates), plasma cortisol levels and expression of behaviours considered to indicate pain, such as restlessness, ear flicking, head shaking, scratching and rubbing (Morisse *et al.* 1995; Heinrich *et al.* 2010; Winder *et al.* 2018), but pain may still occur, especially after the effects of analgesia have worn off (Adcock & Tucker 2018). We also investigate whether there is any evidence of ‘emotional contagion’ when calves observe others being disbudded and whether this is influenced by the observer’s previous experience of disbudding. Previous studies have found evidence for emotional contagion, which may have a significant impact on animal welfare in social species. Goumon and Špinka (2016) found that, while observing conspecifics being restrained, piglets that had previously experienced restraint showed more intense signs of distress than naïve piglets, similarly calves that observed a conspecific suffer from a painful procedure seemed to avoid the location of the procedure even without having experienced it themselves (Ede *et al.* 2019).

In line with the emotional valence hypothesis positing that negative emotions are predominantly processed in the right hemisphere (Leliveld *et al.* 2013), we expected to find changes associated with right-hemisphere activation during situations inducing a negative affective state, i.e. whilst being restrained before administration of local analgesia and NSAIDs prior to disbudding, and once analgesia had worn off the day after. We did not record facial temperature during the disbudding event itself because the heat generated by the process strongly influenced thermal read-outs. In terms of arousal, we predicted that non-lateralised surface temperatures (muzzle, hair whorl) would drop immediately on the disbudding day (D2) during restraint and 3 h after the disbudding compared to the preceding (D1) and following days (D3). For both predictions, we anticipated that calves who had previously experienced disbudding would show stronger effects while watching other calves being disbudded than naïve calves. To test the effect of previous experience, we recorded surface temperatures of calves from three different groups: those that underwent disbudding themselves (Disbudded); those that were present (observing) but did not get disbudded because they had experienced disbudding themselves a few weeks earlier (ExpObs); and those that were present, did not get disbudded, and had never experienced disbudding (InexObs).

Our third and final objective was to examine how face temperatures change as calves approach or move away from a human. According to operational definitions of emotion, (working to) approach and avoid reflect positive and negatively valenced states, respectively (Rolls 2005; Mendl & Paul 2020). Our prediction therefore was that approaching would be associated with lateralised temperature changes indicative of greater left-hemisphere activation and *vice versa* for avoiding.

Arousal was estimated using non-lateralised changes in peripheral temperature in the muzzle, an area studied previously (Proctor & Carder 2015, 2016), and we explored the potential of hair whorl temperature as an additional indicator of arousal as the centre of the hair whorl on the cattle forehead is the only part of the face apart from the muzzle, where there is bare skin not covered by hair which influences thermal read-outs.

## Materials and methods

### Study animals, housing and treatments

Thirty-six female Holstein Friesian calves aged between 20 and 111 days from the University of Bristol’s dairy farm were used for this study. To avoid using sick calves, only individuals that were eating and lying in a normal position, and responsive to their surroundings (moving the ears to stimuli such as vehicles) were selected on the day prior to the onset of recordings. Moreover, the researcher (MAR) liaised closely with the farm vets and informed them about any calves observed to be coughing or having diarrhoea. The vets checked those animals for fever or any other sign of disease and, if they considered the animals to be sick, they were not enrolled in this study. Animals were monitored for signs of illness throughout, but none of the subjects observed for this study were withdrawn.

Routine farm practices involved calves being separated from the dam between one and two days post-birth, after which they were relocated to a semi-open calf shed and kept for two weeks in individual pens (1.0 × 1.5 m; length × width), allowing visual and tactile contact with other calves, and then group-housed in straw-bedded pens (4.8 × 4.1 m) of 6 to 8 calves. Replacement milk was provided for the calves twice a day at 0900 and 1600h. All husbandry procedures complied with UK regulations stated in The Welfare of Farmed Animals (England) Regulations (2007) (SI 2007 No 2078).

Six disbudding sessions were recorded between January and June 2018. These all occurred as part of the farm’s general management procedures (see *Ethical statement*), and the researcher (MAR) joined pre-scheduled farm disbudding sessions to record the thermal videos during the sessions. One week before each disbudding session, the veterinarians checked the health of all calves and selected healthy individuals for disbudding. As part of this study, three groups were created: ‘disbudded calves’ (Disbudded) were calves that went through hot-iron disbudding during the disbudding session; ‘inexperienced observers’ (InexObs) were calves that had not been disbudded, but witnessed the disbudding of another calf, during the current session; ‘experienced observers’ (ExpObs) were calves that had been disbudded in a previous session and witnessed the disbudding of another calf during the current session. In each of the six disbudding sessions, thermal videos were recorded of two calves per group (i.e. six calves per session; 36 in total). The two calves to be ‘disbudded’ were selected by numerical randomisation. The two calves to be ‘inexperienced’ and ‘experienced observers’ were selected on the basis of age, so as to minimise the age difference between the three calf groups. They were the two oldest calves from the InexObs group and the two youngest from the ExpObs group. Calves only participated once in this study, so they could not be allocated to different groups in different sessions. ‘Disbudded’ calves were aged a mean (± SD) of 40.83 (± 7.15) days, ranging from 27 to 52 days old, InexObs were 30.33 (± 13.3), 20 to 64 days old, and ExpObs 74 (± 21.95), 48 to 111 days old.

The hot-iron disbudding sessions were performed between 1400 and 1500h by resident veterinary surgeons accompanied by veterinary students who observed the procedure and assisted with calf restraint. Local anaesthesia via cornual nerve block with procaine (5 ml per bud) and a single intramuscular injection of meloxicam (0.5 mg kg^–1^) was administered 5 min prior to disbudding.

Data were collected for all 36 study calves at two different times: between 1400 and 1500h (T1) and between 1700 and 1800h (T2) on three consecutive days: the day before disbudding (D1); the disbudding day (D2); and the day after disbudding (D3) giving a total of six recordings per calf. The data collected at T1 and T2 on D1 were considered the baselines. T1 was selected as it was the time that disbudding sessions were carried out, and T2 was selected as the time when the local anaesthetic would have worn off on the disbudding day, which is between 45–60 min for procaine (Tranquilli *et al.* 2013).

The following information was collected: ambient temperature (°C) and humidity (%); the calf’s age (days); and the calf’s response to the researcher during the recording (approaching, moving away, not moving, and restricted).

All the procedures and thermal recordings were carried out indoors in the calves’ pens.

### Thermal video data collection

Thermal videos were recorded for 5 (± 2) min for each calf using a FLIR T660^TM^ thermal camera (FLIR Systems Inc, USA), with a resolution of 640 × 480 pixels, a video mode that captured 30 thermal images per second, and a sensitivity of 0.02^o^C and accuracy of ± 1%, focusing on the calf’s head. To extract the temperature data from the regions of interest, hereafter called ROIs (which were selected to represent the eyes, nostrils, nasal passages and ears), the aim of each video-recording session was to capture at least one image from each of the four views selected for this study, which were the front, back, left and right sides of the head. The emissivity of the calves’ skin was fixed at 0.98, in conjunction with previous studies (Montanholi *et al.* 2009; Hoffmann *et al.* 2013; Talukder *et al.* 2014) with the ambient temperature and humidity recorded and updated on the camera every 15 min.

All the thermal videos were recorded while the calves moved freely in the pen, except for the recordings of the calves in the disbudding group on the disbudding day (D2×T1). The experimenter operating the thermal camera remained at a distance between 1 to 2 m throughout all recordings; it was not possible to keep the exact same distance between the researcher and the animal as the animals were moving freely (except on D2×T1). However, previous studies suggest that a difference of 1 m between the thermal camera and the subject has a negligible effect on thermal readings (Fernández-Cuevas *et al.* 2015). The thermal videos from the disbudded calves during the disbudding session (D2×T1) were collected during a standardised restraining process lasting about 2 min prior to the cornual blockage. Care was taken to restrain only the bodies of the calves with two students gently using their bodies to push the calf against a fence, limiting making contact with the calf’s shoulders and legs and avoiding any contact with the head that could affect the temperature of the ROI. The decision to record the reaction of disbudded calves to restraint prior to disbudding as opposed to during disbudding itself was taken since the use of local anaesthetics and contact with the hot iron and/or radiated heat from the hot iron to other parts of the surface of the head, would affect the temperatures recorded (Stewart *et al.* 2008b).

One day prior to data collection (D0), the experimenter visited the pens to select the calves for the experiment and habituate them to the thermal camera by standing in one corner of the pen for 10 min and then moving slowly around the pens pointing the camera towards each calf for 2 min.

On the first recording session (D1×T1), the experimenter entered the first pen and pointed the camera towards the first calf for 2 min prior to starting the recording. The recording was stopped when they considered there were three to four suitable quality frames from each view, i.e. for the side views, frames in which calves had their eyes wide open and at an angle of less than 30° from the camera lens surface; for the front and back views, frames where calves had their ears spread in a symmetrical position, with their head at camera level and less than 30° deviation between the face/head and the camera lens surface. The experimenter then pointed the camera towards the next calf and repeated the procedure until all the calves selected from that pen had been recorded. The same recording order was followed throughout the recording sessions, except for the disbudding session (D2×T1), in which the calves to be disbudded were recorded in the order that the veterinary surgeons and students selected and restrained them.

### Selection of thermal images for analysis

The thermal videos were blinded to the researcher by assigning random numbers as identification, and were examined using FLIR Tools software (Teledyne FLIR, USA), and frames considered to be good quality exemplars of each view were extracted. This was followed by selection of the best frame for each directional view in each thermal video, according to the following criteria: for the front and back views, images with the calf’s ears fully extended and in the most symmetrical position were selected; for the left and right views, the most identical images judged by eye from both sides of the head in terms of eye position (eye opening and iris location) and camera position (distance and angle from the camera) were selected. In all cases, images where the plane of the surface of interest was oriented ≥ 30^o^ away from the surface of the camera lens, and images collected immediately after drinking or external disturbances (e.g. vehicles moving inside the building or staff moving around) were not used. One frame per time-point per view was selected, as analysis of the influence of different factors (time within recordings, camera angle, camera elevation, etc) on the baseline thermal data indicated that as long as images used to calculate temperature asymmetries shared similar camera angle and elevation, only a small proportion of the variance between images could be attributed to time differences between images. For example, when looking at images from the baseline of this study, a difference of 0.12°C in a 2-min period can be found in temperature asymmetries from the ocular (inner corner of the eye), periocular (caudal eye surrounding) and nasal airway areas (Ramirez Montes De Oca 2021).

**Figure 1.**
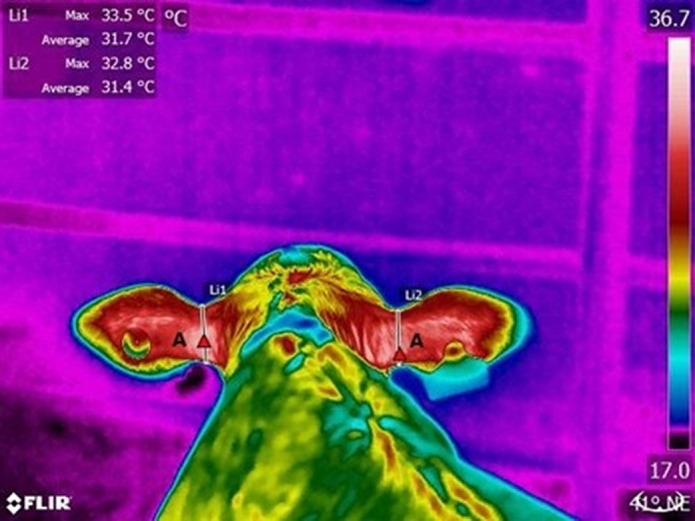
Delimitation used for temperature data collection from thermograms of the back view of the calves (n = 36), showing the ear base areas (A).

**Figure 2.**
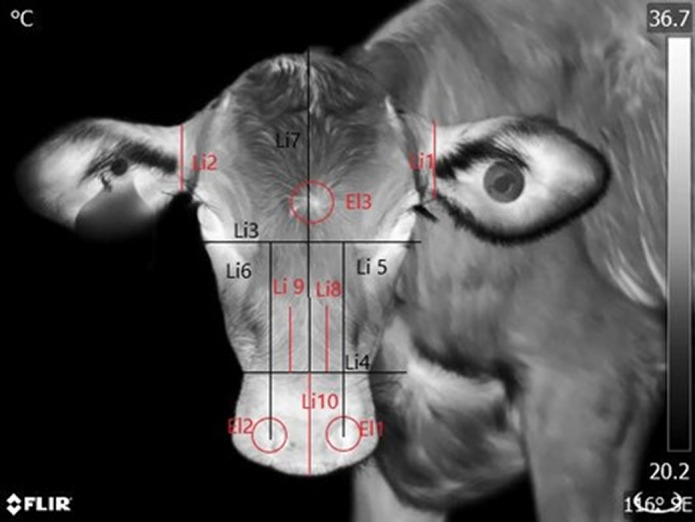
Delimitation used for temperature data collection from thermograms of the front view of the calves (n = 36); showing the ears (Li1 and Li2), nostrils (El1 and El2), nasal passages (Li8 and Li9), hair whorl (El3) and muzzle areas (Li10).

**Figure 3.**
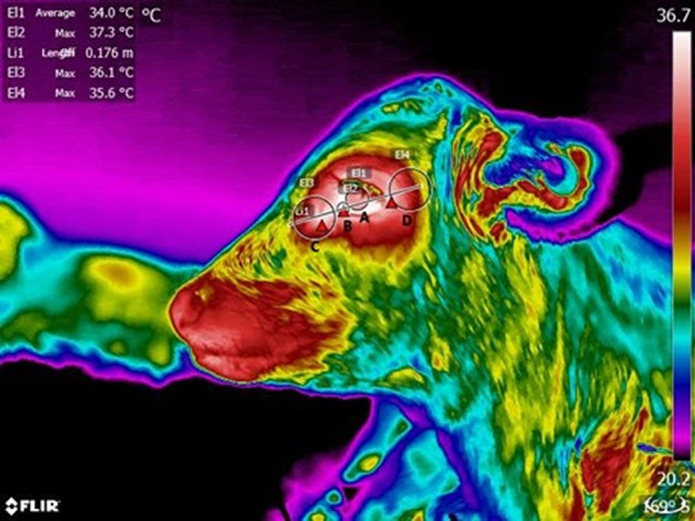
Delimitation used for temperature data collection from thermograms of the side views of the calves (n = 36); showing the eyeball (A), inner corner (B), rostral eye surrounding (C) and caudal eye surrounding areas (D).

Once selected, the file names of the images were replaced by randomised numbers to blind researchers to their identity during the measuring process. For each recording, the calf’s behavioural response to the researcher was also scored. The response of the calf to the researcher was scored according to the number of times that the animal approached the researcher or moved away from him); movements to interact with conspecifics or to reach the drinker/feeder were not included. For example, if during a recording the calf scored a higher number of approaches than moving away, then the calf was scored as ‘approaching’; if calves moved away more than they approached, they were scored as ‘moving away’, and if calves did not move in response to the researcher, they were scored as ‘not moving’. All Disbudded calves were recorded as ‘restrained’ during the disbudding session (D2×T1). Table 1 summarises the temperatures extracted from the different ROIs. Figures 1, 2 and 3 illustrate these ROIs.

**Table 1.** Regions of Interest (ROI) for each of the camera views and types of temperature data collected in the dairy calves from this study (n = 36 calves × 6 time-points = 216 images per view)

Camera View	ROIs	Temperatures °C extracted	Data points used in the analysis:
Back	Ear base	Maximum and average	Asymmetry
Front	Ear base	Maximum and average	Asymmetry
	Nostrils	Maximum and average	Asymmetry
	Nasal passages	Maximum and average	Asymmetry
	Muzzle	Maximum and average	Raw data
	Hair whorl	Maximum	Raw data
Left / right	Eyeball	Average	Asymmetry
	Inner corner	Maximum	Asymmetry
	Rostral eye surrounding	Maximum	Asymmetry
	Caudal eye surrounding	Maximum	Asymmetry

’Maximum’ refers to the pixel with the highest surface temperature within the ROI, and ’average’ refers to the average surface temperature of the ROI. Data points were of two types: either raw temperature data extracted from the image or the temperature asymmetry between two ROIs expressed as left-side temperature minus right-side temperature.

### Measurements of the ROIs

Once images for each of the four views (front, back, left and right) were selected for analysis, FLIR Tools+ software was used to delimit and extract the temperatures of the different ROIs. For maximum temperatures, only the warmest pixel was used, whereas average temperatures were calculated using all the pixels within the delimited area. All the lines used to delimit the ROIs had a width of 1 pixel.

#### Back view: Base of the ears (Figure 1)

The pixels used to measure the temperature of the left and right ear bases were those lying within narrow straight lines (Li1, Li2) drawn between the lower point on the superior curve of the ear to the upper point of the inferior curve of the ear as shown in Figure 1.

#### Front view: Ears, nostrils, nasal passages, muzzle and hair whorl (Figure 2)

To delimit the pixels used to calculate the temperatures of the frontal ear base areas, a straight line from the lower part of the upper curve of the ears towards the highest point of the lower curve of the ears was drawn (Li1 and Li2). Nostril areas were delimited by circles with radii extending from the centre of each nostril towards the furthest visible point of the upper rim of the nostril (El1 and El2). To delimit the area of the nasal passages, a horizontal line was drawn from one caruncle to the other (Li3), followed by a line parallel to Li3 at the top of the muzzle (Li4). Two vertical lines (Li5 and Li6) were drawn from the centre of the nostrils (El1 and El2) towards Li3. Then, a vertical line (Li7) was drawn in between Li5 and Li6, from Li4 to the top of the head. Finally, the areas encompassing pixels in the nasal passage used to measure nasal temperature were defined by two narrow vertical lines (Li8 and Li9) in the spaces between Li5 and Li7, and Li6 and Li7. Those lines extended up from Li4 and were half the length of Li5 and Li6. Pixels for measuring muzzle temperatures (Li10) lay within a vertical line from Li4 to the bottom of the muzzle centrally located between the two nostrils (an extension of Li7). The hair whorl is the hairless area in between the eyes (observed as the warmest point in the forehead), measured within the pixels contained in a circle (El3) with a radius extending from the centre of the whorl to its furthest visible point (hairless area). Measures are illustrated in Figure 2.

#### Left and right views: Eye and surrounding areas (Figure 3)

The area of the eyeball was delimited drawing a circle (El1) with a radius extending from the centre of each eyeball to the edges of the iris. The inner corner of the eye was delimited by a circle (El2) half the diameter of El1 centred on the inner canthus of the eye. To measure the rostral eye surrounding area, a line (Li1) was drawn from the inner canthus to the outer canthus of the eye, extending the line outside each canthus for a distance representing 5 cm in real life. A circle (El3) with a radius equivalent to the distance from the centre of the iris to the inner corner was drawn. Finally, the centre of El3 was moved along Li1 to a length equivalent to the radius of El1 in front of the eye. This defined the rostral eye surrounding area. The caudal eye surrounding area was a circle (El4) equivalent in size to El3 with its centre located on Li1 at a distance equal to the radius of El1 from the eyelashes of the calf (see Figure 3).

### Statistical analysis

For the ROIs used to study whether lateral differences in temperature are linked to affective valence, the differences between the left and right temperatures (left minus right) were calculated; positive values indicate higher temperatures on the left side and negative values higher temperatures on the right. Raw temperature data were used to infer arousal levels and were taken from non-lateralised, single regions (muzzle and hair whorl).

### Correlations between temperatures in different ROIs

A correlation matrix controlling for the effect of ‘calf ID’ between the 15 ROIs was created to identify ROIs that provided similar information (210 correlations). To control for multiple tests, the Benjamin-Hochberg procedure to control for a False Discovery Rate of 0.2 was carried out using all the *P*-values. The ROI whose temperature readings were most strongly correlated with those of other ROIs in the same area (ears, nasal and ocular/periocular regions) was selected as the primary temperature measurement site for that area. The ROI that was least correlated with it, and hence might offer relatively independent information about that area, was selected as the second temperature measurement for further analysis (see Table 2). For example, in the ocular/periocular areas, inner corner of the eye was selected as it was significantly correlated with all the other areas (eyeball, caudal and rostral eye surrounding areas). Caudal eye surrounding area was then selected as it was the least correlated with the inner corner of the eye measure (see Table 2).The exception to this rule was between muzzle and whorl temperatures, in which all temperatures were used as we were looking at the suitability of hair whorl temperatures as an alternative to muzzle temperatures.

**Table 2.** Main correlations between temperature asymmetries from the different measurements within each area (Ear, ocular/periocular and nostril/nasal passages), data from 216 images (n = 36 dairy calves × 6 recording sessions)

Ear area		
ROI (measurement)		Correlation
Ear base front (max) L–R*	Ear base front (ave) L–R	r = 0.785, *P*<0.001
	Ear base back (ave) L–R	r = 0.436, *P*<0.001
	Ear base back (max) L–R**	r = 0.394, *P*<0.001
Ocular / periocular area		
ROI (measurement)		Correlation
Inner corner of the eye (max) L–R*	Rostral eye surrounding (max) L–R	r = 0.518, *P*<0.001
	Eyeball (ave) L–R	r = 0.248, *P*<0.001
	Caudal eye surrounding (max) L–R **	r = 0.181, *P* = 0.008
Nostrils / nasal passages		
ROI (measurement)		Correlation
Nostril (ave) L–R*	Nostril (max) L–R	r = 0.770, *P*<0.001
	Nasal passage (ave) L–R	r = 0.573, *P*<0.001
	Nasal passage (max) L–R**	r = 0.551, *P*<0.001

The abbreviation L-R means the temperature asymmetry is expressed as the left-side temperature minus the right-side temperature. The abbreviation (max) means the data-point is the highest temperature of any pixel within the delimited region of interest, and (ave) means the data-point is the average temperature across the delimited region of interest. *These measurements were selected for further analysis as they had the highest correlations with other ROIs within each area **These measurements were selected for further analysis as they had the lowest correlation with the first ROI selected within each area.

### Effects of predictor variables on facial temperatures at specific time-points

Statistical models were built using the data from all six recording sessions to identify significant associations between temperature (temperature asymmetries in bilateral areas [L-R], or muzzle and whorl temperatures) and potential predictor variables: THI (Temperature-Humidity Index calculated as: 0.8 × T + RH × (T–14.4) + 46.4, in which ‘T’ is ambient temperature in °C and RH relative humidity, expressed in decimals); calf age; if calves were restrained (Yes or No); Day (D1, D2, D3); Time (T1, T2); and the Day × Time interaction; calf group (InexObs, Disbudded, ExpObs) and response of the calves to the camera (approaching, moving away, not moving and restrained). To account for repeated measures, linear multilevel models were created by including calf as a random effect (six recordings per calf). To create these models, bivariable correlations were first examined: each predictor variable (calf age, calf group, restraint, Day, Time, response to the researcher or THI) was tested individually (*P*-value) with the outcome variable (temperature asymmetries or raw temperature [in muzzle and hair whorl areas]). In addition, Day and Time were tested together, with an interaction term (Day × Time) to account for the specific conditions at the six time-points (recordings). *P*-values were calculated using likelihood-ratio tests. For each outcome variable, the explanatory variable with strongest association (based on the likelihood-ratio test) was retained in the model and final models were created by a process of forwards regression. Each of the remaining variables were added one at a time, beginning with the strongest association; significant variables were retained until no further improvement of the model was found. Final models were those in which all variables were significant (*P* < 0.05), e.g*. “Inner corner max (Left-Right) D2 × T1_ij_ = β_oji_ constant + Variable A_ij_* + *Variable B_ij_ +…”.* For each model, normality was confirmed by visual examination of normality and caterpillar plots of the standardised residuals × the normal scores), no transformations were required as all the residuals from the models showed a normal distribution.

#### Effects of calf group on facial temperatures by recording session

As we found no significant association between facial temperature and calf group, further analyses were carried out to investigate main differences between groups at each time-point. We first calculated the means and 95% confidence intervals of asymmetric differences and muzzle and whorl temperatures for each calf group at each data collection time-point. Visual inspection of these values plotted in Microsoft Excel® version 16.0 allowed us to identify those time-points at which the confidence intervals of two or more of the groups did not overlap. Simple regression models (with one data-point per calf) were then built for each of the ROIs identified using data from that time-point and adding variables in a stepwise regression (as above but excluding time-point, Time and Day as variables).

All statistical models were carried out using the multilevel modelling software MLwiN v3.00 (http://www.bristol.ac.uk/cmm/software/mlwin/). To control for multiple hypotheses, the Benjamin-Hochberg procedure to control for a False Discovery Rate of 0.2 was carried out using all the *P*-values calculated from the likelihood-ratio tests between the base models and the models, including the single variables (McDonald 2009).

### Ethical statement

This research was approved by the University of Bristol Animal Welfare and Ethical Review Body. All the husbandry procedures during the data collection period were performed in accordance with UK legislation as stated in The Welfare of Farmed Animals (England) Regulations 2007 (SI 2007 No 2078). This study did not alter any of timings or routines as regards normal daily farm activities, but simply made thermal recordings of the procedures as described above.

## Results

In presenting results, we provide tables of final model coefficients, *P*-values and directions of effects in the Supplementary material. An exemplar table is provided for the first set of results presented (Table 3).

**Table 3.** Exemplar table of a final model showing the estimated coefficients for asymmetries in the maximum temperature of the Inner corner of the eyes (L-R) calculated using linear multilevel models (n = 36 calves × 6 recording sessions; 216 images in total), showing the effect of reaction to the researcher (df = 3), using “not moving” as the reference category

Variable	Categories	Coefficient	S.E.	p-value	Asymmetries towards	Active hemisphere (hypothesised)
Constant	n/a	–0.026	0.056	*P* = 0.649		
Reaction to the researcher	Moving away	0.167	0.126	*P* = 0.187		
	Approaching	–0.341	–0.613	*P* = 0.014	Right eye	Left hemisphere
	No movement					
	Restrained calves on the disbudding session	–0.106	0.180	*P* = 0.555		

Tables for the rest of the models can be found in the Supplementary material.

### Correlations between temperatures in different ROIs

The maximum and average temperature asymmetries within ROIs were highly correlated (n = 36 calves, 216 images per view) (*r* = 0.770 to 0.872; *P* < 0.001). Muzzle and hair whorl areas had high correlation values (*r* = 0.593 to 0.701; *P* < 0.001) and temperature asymmetries in ROIs of areas close to each other were moderately correlated (*r* = 0.248 to 0.518; *P* < 0.001), except for caudal eye surrounding with eyeball (*r* = 0.068; *P* = 0.320) and with rostral eye surrounding (*r* = 0.041; *P* = 0.551), see Table 2 for all significant correlations and the measures that were selected for further statistical analyses on temperature asymmetries (indicated by asterisks).

### Effects of predictor variables on facial temperatures

**Figure 4.**
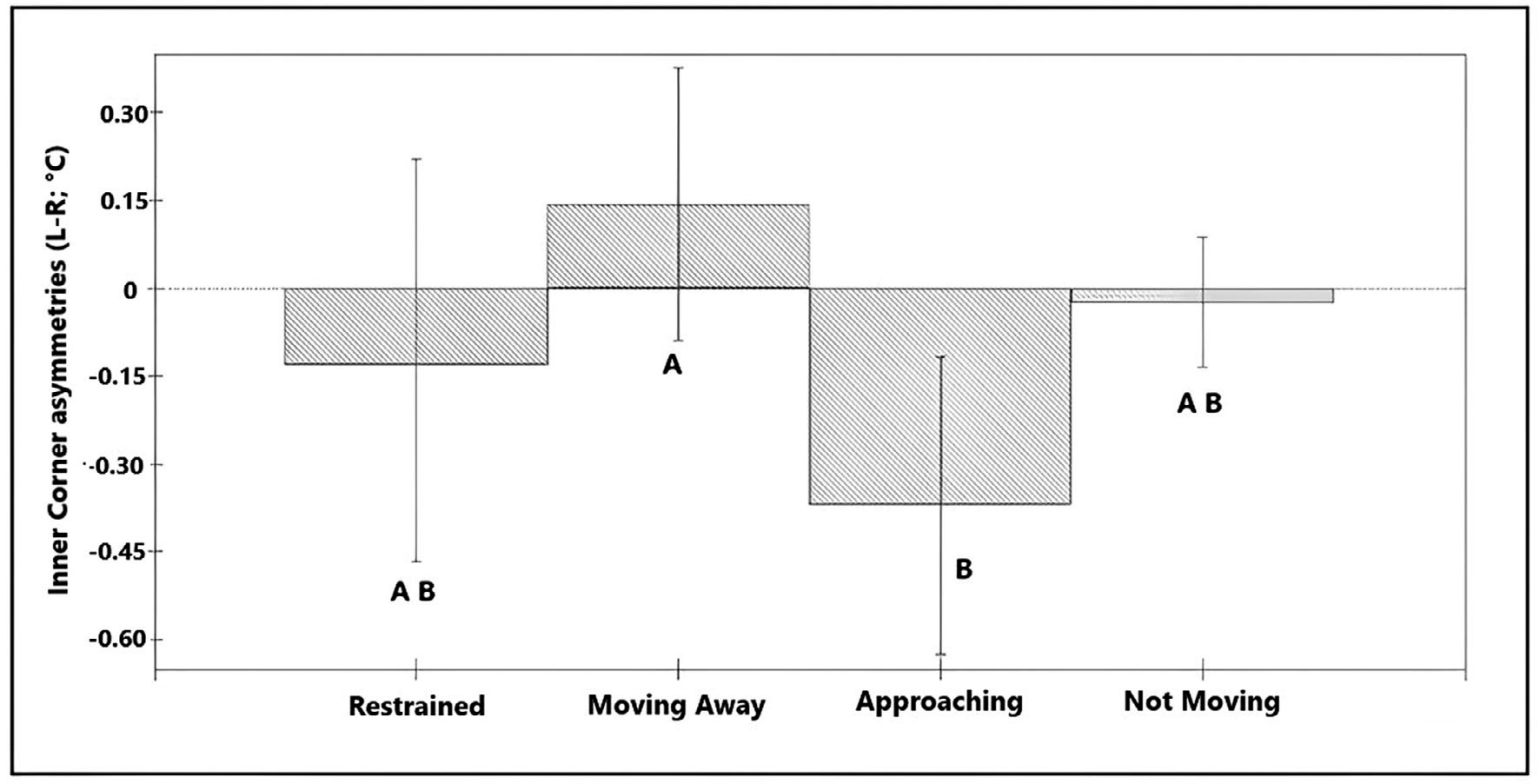
Bar chart showing the predicted values for the different responses of the calves to the researchers, based on the final model for temperature asymmetries in the inner corner of the eye (L-R) with the full data-set (36 calves × 6 recording sessions = 216 images per side) * Bars showing different superscripts differ significantly from each other (*P* < 0.05).

Asymmetries in the temperature of the inner corner of the eye (L-R) (n = 36 calves × 6 recording sessions; 216 images in total) were associated with differences in the reaction of the calf towards the researcher (χ^2^ = 8.73, df =3; *P* = 0.033; Table 3) explaining 4.7% of the total variance in the data. On average, temperature asymmetries in the inner corner of the eye were higher towards the right eye in calves that reacted by approaching the researcher (L-R was negative) compared to calves that moved away. Calves that did not move in response to the researcher tended to have warmer left eyes (L-R was more positive) but did not show significant asymmetries (Figure 4). Restraint during the disbudding session was not associated with a significant asymmetry in inner corner temperatures. Asymmetries in the caudal eye surrounding area did not show a significant association with any of the variables studied (*P* > 0.05).

Tables for the rest of the models can be found in the Supplementary material.

**Figure 5.**
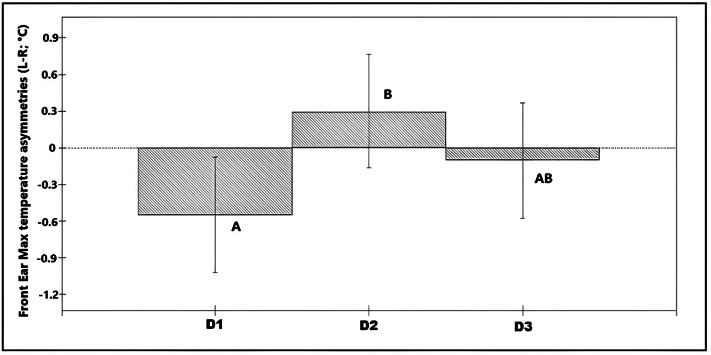
Bar chart showing the predicted values for the different observation days, based on the final model for asymmetries in the maximum temperature of the ear (front view) (n = 36 calves × 6 recording sessions; 216 images in total). * Different superscripts denote significant differences (*P* < 0.05).

Asymmetries in maximum values of the front ear temperature (n = 36 calves × 6 recording sessions; 216 images in total) were associated with day of recording (χ^2^ = 6.88, df = 2; *P* = 0.032; Table S1 in Supplementary material) explaining 3.3% of the total variance on the data. On average, temperature asymmetries in the front of the ear were higher towards the left ear in D2 (L-R was positive) compared with asymmetries in Day 1 in which the right ear was significantly warmer (L-R was negative; Figure 5). Asymmetries in nostril average temperatures and maximum temperatures from the back of the ears showed no significant association with any of the predictor variables (*P* > 0.05).

The maximum temperature of the muzzle (n = 36 calves × 6 recording sessions; 216 images in total) increased with THI (χ^2^ = 62.07, df =1; *P* < 0.001; Table S2 in Supplementary material) explaining 38.2% of the total variance on the data. The average temperature of the muzzle increased with THI (χ^2^ = 76.63, df =1; *P* < 0.001; Table S3 in Supplementary material) explaining 44.2% of the total variance on the data. The maximum temperature of the hair whorl increased with THI (χ^2^ = 75.32, df = 1; *P* < 0.001; Table S4 in Supplementary material) explaining 49.6% of the total variance in the data.

### Effects of calf group on facial temperatures at specific time-points

Although calf group (InexObs, Disbudded, ExpObs) was not significant in any of the models above, examination of means and 95% confidence intervals indicated potential differences between groups at two time-points (D2×T1; D3×T1), thus they were examined separately.

**Figure 6.**
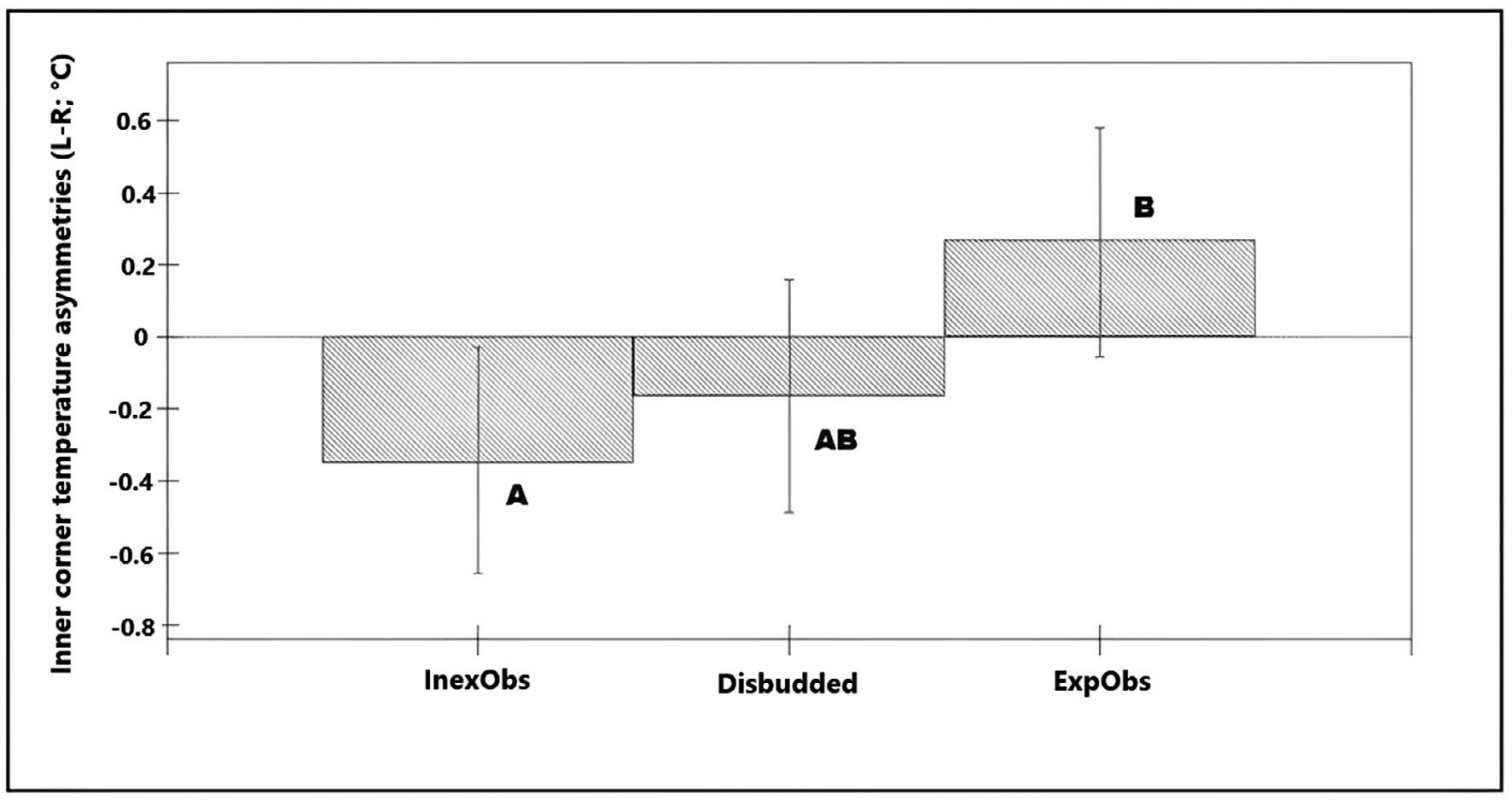
Bar chart showing the predicted values for the different calf groups, based on the final model for temperature asymmetries in the inner corner of the eye (L-R) during the disbudding session (D2×T1) (n= 36 calves × 1 recording). * Bars showing different superscripts differ significantly from each other (*P* < 0.05).

Inner corner eye asymmetries in the disbudding session during which calves were restrained (D2×T1) (n = 36 calves × 1 recording) were associated with calf group (χ^2^ = 6.82, df = 2; *P* = 0.033; Table S5 in Supplementary material), explaining 17.3% of the variance in the data. Calves in the ExpObs group had significantly larger magnitude asymmetries towards the left eye (L-R was positive) than calves in the InexObs group (L-R was negative; Figure 6).

**Figure 7.**
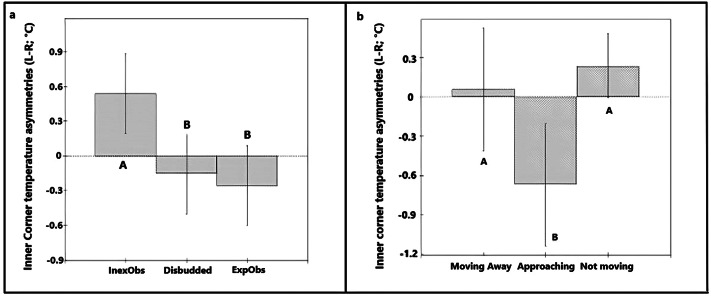
Bar chart showing the predicted values for: (a) calf groups and (b) response to the researcher, based on the final models for temperature asymmetries in the inner corner of the eye (L-R) on the day after disbudding (D3) at T1 (1400–1500h) (D3×T1) (n = 36 calves × 1 recording).

Inner corner eye asymmetries on the day after disbudding (D3×T1) (n = 36 calves × 1 recording) were associated with calf group (χ^2^ = 9.76, df = 2; *P* = 0.007) and the response of calves to the researcher (χ^2^ = 9.09, df = 2; *P* = 0.010; Table S6 in Supplementary material), explaining 39.1% of the total variance in the data. Calves in the InexObs group had temperature asymmetries towards the left eye (L-R is positive) in comparison to calves from the Disbudding and ExpObs group which had asymmetries towards the right eye (L-R is negative; Figure 7[a]). Calves that approached the researcher during the recording period had asymmetries towards the right eye (L-R is negative) compared with calves that moved away or did not move in response to the researcher, which had asymmetries towards the left eye (L-R is positive; Figure 7[b]]).

## Discussion

This study aimed to explore whether asymmetries in calf facial temperature in different regions of interest (ROIs) exist and, if so, whether the direction of those asymmetries reflect enhanced right brain hemisphere activity predicted by the emotional valence lateralisation hypothesis to occur in negative affective states. In this study, we assumed such states to be associated with restraint for disbudding, response to others being disbudded, and avoidance of humans. Our key findings were that: calves from all groups had warmer left ears on the day of disbudding, whereas the opposite was the case on the previous day; calves that had experienced disbudding had higher left eye temperatures when observing other calves being disbudded compared to inexperienced calves; calves that avoided the researcher had warmer left eyes whilst those approaching them had warmer right eyes. These findings may indicate greater right hemispheric activity in those animals with left-biased elevated temperatures which may, in turn, reflect a more negative affective state. However, there are a number of caveats to our findings in terms of limitations of this exploratory study, and we start by considering these before discussing the findings themselves.

One limitation was the relatively small number of animals (n = 12 calves per group) and the lack of previous data to guide estimation of suitable sample sizes. This, coupled with the complexity of the cross-time changes in calf experience (modelled by Day × Treatment interactions), may have prevented the detection of robust effects. For example, putative factors influencing thermal responses during D2×T1 included response to restraint and different observer experience, in contrast, experience of pain was more likely to be a predominating influence on thermal response during D3, and on D1 responses to the relatively neutral (baseline) situation were more likely influenced by the presence of the researcher/camera.

Another limitation of this study was age variation between groups, resulting from the need to follow the farm’s husbandry protocols that prevented selection of calves of specific ages for each treatment group. We tried to reduce the variation by selecting older calves for the inexperienced observer group and youngest from the experienced observer group. However, the experienced observer group was consistently older and with higher age variability. Consequently, we tested for effects of age as an explanatory variable, but no significant effect was detected, and this variable was not retained in any of the final models.

A third limitation was the restricted number of images that could be analysed as exemplars of each particular day/time-point due to the time-consuming nature of by-eye coding and analysis. We approached the issue of image selection in a systematic way, but automated extraction of specific ROI data from thermal images would significantly enhance the practicability, reliability, and potential of this approach.

### Ear temperature asymmetries

Innervation of the ears is considered to come from both ipsilateral and contralateral hemispheres, albeit with significantly more afferent nerves coming from the contralateral hemisphere (Budras & Berg 2011). This suggests that temperature changes due to increased blood flow or muscular activity, are more likely to reflect higher activation of the contralateral hemisphere. Considering the data from all calves, we found higher asymmetries towards the left ear on the disbudding day (D2) compared to those observed in the baseline recordings (D1). Following the above rationale this suggests a higher activation of the right hemisphere on the disbudding day (D2) which, in line with the emotional valence lateralisation hypothesis, may indicate a more negative state on that day than during the baseline recording. On D1, a baseline asymmetry towards the right ear/left hemisphere was found which could be due to a generally more positive reaction to humans (i.e. food provision). It is important to highlight that these temperature asymmetries were observed only in the front images. A couple of possible explanations include that the delimitation of our measurements from the back ear was not accurate enough, or that mechanisms involved in temperature changes between the areas measured in the front and back images differed.

### Eye temperature asymmetries

As discussed in the *Introduction*, temperature changes in the eye area are likely to reflect activity of the contralateral hemisphere. We hypothesised significant differences in eye temperature asymmetries between the Disbudded calves and the Observer groups on the disbudding day (D2) and on the day after (D3). However, those differences were not found. During the disbudding session (D2×T1), we expected to see changes associated with higher activity of the right hemisphere as a response to physical restraint, but this was not observed, perhaps because calves differed in their behaviour while restrained (Marco Ramírez, personal observation 2018), which could indicate that some found the experience less aversive than others. Differences in responses to handling can be associated with temperament, fearfulness and reactivity to novelty, as well as previous negative or positive experiences (Grandin & Shivley 2015).

On the day after disbudding (D3), changes suggesting right-hemisphere dominance can be expected in the disbudded calves if residual pain caused by the procedure was present on that day, but those were not observed in our data. It is possible that because of analgesia received in the aftermath of the procedure, calves were not in sufficient pain to exhibit the asymmetric changes expected or that we did not find significant effects in our data due to the study’s sample size. Some studies of calf behaviour and cortisol levels have found meloxicam to have a significant effect on the reduction of pain behaviours and that its effects may last up to five days after disbudding (Stafford & Mellor 2005; Dockweiler *et al.* 2013). However, we cannot be sure that the calves were not in pain, because other evidence points to long-lasting effects of disbudding even with the use of analgesics, including pain behaviour being expressed on days following the disbudding event (for a review, see Herskin & Nielsen 2018).

The previous experience of calves influenced the direction of lateralised differences in eye temperature during the disbudding session. Asymmetries in the inner corner of the eye showed higher temperatures in the left eye of Experienced Observers and right eye of Inexperienced Observers during the disbudding session (D2×T1), suggesting that calves with experience of disbudding showed greater right hemisphere activation. The emotion valence lateralisation hypothesis would thus indicate that they processed the disbudding of conspecifics as a more threatening or negative situation relative to Inexperienced Observers. It is possible that Inexperienced Observers perceived the situation as something positive as they did not realise what the situation represented, having not experienced it themselves. This aligns with the findings of Goumon and Špinka (2016) that piglets who had had a negative experience (being restrained) behaved more fearfully than inexperienced piglets when observing a conspecific being restrained.

Changes in eye temperature on the day after disbudding (D3×T1) showed the reverse pattern with Experienced Observers having warmer right eyes and Inexperienced Observers warmer left eyes, suggesting a left hemisphere activation of the former and right hemisphere activation in the latter. A speculative explanation is that on the day after disbudding, the situation did not resemble a disbudding session for those calves who had experienced two such occasions (Experienced Observers) since other calves were behaving calmly, and olfactory stimuli such as gas or burned tissue were absent. On the other hand, the Inexperienced Observers with only one experience of the commotion and potential emotional contagion of a disbudding event may have more readily associated this with the presence of the researcher and hence shown an anticipatory negative response when the researcher returned.

Previous research has shown that cows can associate previous experiences with specific people and specific places or situations (de Passillé *et al.* 1996; Munksgaard *et al.* 2001; Ede *et al.* 2019) For example, de Passillé *et al.* (1996) found that calves learned to discriminate between an aversive and a positive handler wearing different clothes, showing fewer interactions with the negative handler after repeated exposures, especially in the place where the aversive treatment was carried out. Munksgaard *et al.* (2001) found that cows observing a handler treating a conspecific negatively learned to avoid the handler without experiencing the negative handling themselves. Similarly, Ede and colleagues (2019) found that disbudded calves are less likely to lie down and spend less time in the area where their disbudding occurred. Differences between our observer groups could thus be related to previous experience.

The finding that calves that approached the researcher/camera had warmer right inner eye corners than calves that moved away, could indicate activation of the left hemisphere in the former animals in line with the approach withdrawal hypothesis that approach behaviour is more closely associated with left hemisphere activation.

### Nostril temperature asymmetries

The muscles responsible for dilation and contraction of the nostrils are innervated by the buccal branch of the facial nerve, which is considered to be ipsilateral in its origin, hence our hypothesis that asymmetries in nostril temperatures might reflect higher activity of the ipsilateral hemisphere. However, none of the predictor variables in this study significantly influenced the direction of temperature asymmetries.

### Whorl and muzzle temperatures

In terms of (non-lateralised) arousal indicators, muzzle temperatures and hair whorl temperatures did not differ significantly between recordings, days or calf groups, suggesting that the different affective responses elicited during the study may have not caused a significant change in arousal as observed in previous experiments. Previous experiments looking at muzzle temperature have monitored the temperature changes in a more continuous way and during a shorter period of time, due to our design we just use one image per situation which could have led us to miss any changes in this area (Kuraoka & Nakamura 2011; Proctor & Carder 2016).

In practice, the muzzle area might not be the most appropriate area to measure temperature, as it can be affected by changes in breathing patterns and is in continuous contact with external factors such as food and water. We found a high correlation between muzzle and whorl temperatures and similar patterns through the different recording sessions. Therefore, we suggest temperature of the hair whorl as a potential alternative to muzzle temperature in dairy cattle.

In our study, we found that the ambient temperature-humidity index (THI) showed a linear relationship with muzzle and hair whorl temperatures, likely mediated by thermoregulatory mechanisms. It is important to highlight that THI values found in this study (between 48 to 70) are considered to be within the thermal comfort zone for calves (Fuquay 1981; Lim *et al.* 2021) and the relationship between calf skin temperature and THI levels above 75 might differ as a result of thermal stress.

## Animal welfare implications and Conclusion

To our knowledge, our study is the first to investigate asymmetries in ear and eye temperatures of dairy calves and find some associations between these and calf experience and responsiveness to humans. Our initial findings suggest that asymmetries in skin surface temperatures may be a useful parameter to consider in developing new non-invasive physiological markers of animal welfare. Moreover, they indicate that ocular and ear regions are more promising cow facial areas for further investigation than nostril and nasal passage structures, and that the inner corner of the eye seems to be the most interesting area for further exploration of those asymmetries. There thus seems to be potential for the use of infrared thermography as a non-invasive tool for detecting temperature changes that are not only influenced by affective arousal but also, and importantly, by affective valence. However, it is essential to highlight that this study only provided initial evidence of temperature asymmetries under this context. To fully understand the physiological mechanisms underlying asymmetries in skin surface temperature and their relationship with brain lateralisation, a substantial amount of fundamental research and subsequent validation of this method with behavioural and physiological parameters like EEG, HRV and behavioural correlates, will be required. We also acknowledge that several ROIs were explored in this study and that results differed between those regions. This may be due to small sample sizes, or it may reflect a genuine mechanistic difference in any lateralised links between brain activity and surface temperature in different areas of the face. As part of the main objective was to identify which areas might provide more information regarding temperature asymmetries in the context of animal emotions, these findings provide the basis for further work.

## Supporting information

Ramirez Montes de Oca et al. supplementary material Ramirez Montes de Oca et al. supplementary material

## Supplementary material

The supplementary material for this article can be found at http://doi.org/10.1017/awf.2024.47.
