# Supplementary material for: An exploration of surface temperature asymmetries as potential markers of affective states in calves experiencing or observing disbudding
Source: Anim Welf. 2024 Nov 5;33:e45. doi: 10.1017/awf.2024.47 (PMC11589073; doi:10.1017/awf.2024.47)
Supplement: Ramirez Montes de Oca et al. supplementary material [file S0962728624000472sup001.pdf]

Table S1. Final model for asymmetries in Front Ear max temperature (L-R)

Variable	Categories	coefficient	S.E.	*P*-value	Asymmetries towards	Active hemisphere (hypothesised)
Constant	n/a	0.584	0.2829	*P*=0.039		
Recording day	Disbudding day (D2)	Reference category				
	Day before disbudding session (D1)	-0.850	0.322	*P*=0.005	Right ear	Left hemisphere
	Day after disbudding session (D3)	-0.381	0.322	*P*=0.214		

Table S2. Final model for Muzzle maximum temperature

Variable	Categories	Mean	S.E.	*P*-value
Constant		32.994	0.177	*P*<0.001
THI	n/a	0.227	0.024	*P*<0.001

Table S3. Final model for Muzzle average temperature

Variable	Categories	Mean	S.E.	*P*-value
Constant		29.749	0.307	*P*<0.001
THI	n/a	0.418	0.040	*P*<0.001

Table S4. Final model for Hair Whorl max temperature

**Variable**	**Categories**	**Coefficient**	**S.E.**	***P*-value**
Constant		30.523	0.266	*P*<0.001
THI	n/a	0.335	0.032	*P*<0.001

Table S5. Final model for Inner Corner Eye max temperature (L-R) in the disbudding session (D2 x T1)

**Variable**	**Categories**	**Mean**	**S.E.**	***P*-value**	**Asymmetries towards**	**Active hemisphere (hypothesised)**
Constant	n/a	-0.081	0.105	*P*= 0.444		
Calf group	InexObs	Reference category				
	Disbudded	0.183	0.235	*P*= 0.434		
	ExpObs	0.625	0.235	*P*= 0.008	Left eye	Right hemisphere

Table S6. Final model for Inner Corner Eye max temperature (L-R) on D3 x T1

**Variable**	**Categories**	**Coefficient**	**S.E.**	***P*-value**	**Asymmetries towards**	**Active hemisphere (hypothesised)**
Constant	n/a	0.534	0.246	*P*=0.030		
Calf response to the camera	Move away	Reference category				
	Approach	-0.707	0.336	*P*=0.035	Right eye	Left hemisphere
	Not moving	0.182	0.274	*P*=0.507		
Calf group	InexObs	Reference category				
	Disbudded	-0.680	0.246	*P*= 0.006	Right eye	Left hemisphere
	ExpObs	-0.793	0.260	*P*= 0.002	Right eye	Left hemisphere
